# Physical activity and Alzheimer’s disease: a call for evidence incorporation

**DOI:** 10.1016/j.tjpad.2025.100225

**Published:** 2025-06-07

**Authors:** Juan Luis Sánchez-Sánchez, Philipe de Souto Barreto

**Affiliations:** a IHU HealthAge, Toulouse, France; b Institut du Vieillissement, Gérontopôle de Toulouse, Centre Hospitalo-Universitaire de Toulouse, Toulouse, France; c CERPOP UMR 1295, University of Toulouse III, Inserm, UPS, Toulouse, France

**Keywords:** Physical exercise, Alzheimer disease

Life expectancy has increased worldwide in the last decades, leading to higher rates of chronic conditions, including neurodegenerative diseases. Alzheimer’s disease (AD) is the leading cause of cognitive impairment among older adults. It affects around 5 %, 13 % and one third of people in the 65–74, 75–84 and >85 age groups, respectively [1]. According to the canonical pathogenesis theory of AD, known as “amyloid cascade”, the condition is characterized by the brain accumulation of extracellular β-amyloid plaques and intracellular tau neurofibrillary tangles, and is often accompanied by neuroinflammation and neurodegeneration [2]. However, accumulating evidence highlights that non-familial (i.e. age-related AD) has a multifactorial nature, with defective proteostasis and elimination of cellular “waste” processes, immune response and mitochondrial dysfunction, and neuroinflammation playing an important role. Notably, these mechanisms are core hallmarks of the aging processes. Together with evidence of AD pathophysiological features in non-demented older adults, this calls for the need to incorporate evidence on the role of the biology of aging in AD.

The incorporation of biology of aging insights might assist in the understanding of how the brain aging process might contribute to dementia development in the scope of AD. Geroscience, a field of knowledge devoted to investigate the genetic, molecular, and cellular mechanisms that make aging a major risk factor and driver of common chronic conditions and diseases of older people, might be a fundamental ally in this regard. Under the Geroscience principles, the intervention on mechanisms involved in biological aging would assist in the prevention or delay of chronic conditions, including AD-related dementia [3].

Lifestyle-based interventions targeting modifiable factors associated with AD are deemed the most effective strategies for disease prevention. Among them, physical activity (PA) outstands as a protective factor against the onset of AD-related dementia and cognitive decline in those with prevalent disease. Emerging evidence also points that the association of PA with cognitive health at older age might happen through the modulation of some of the core physio-pathological changes associated with the disease (inflammation, impaired Aβ clearance, cell death, lipid dyshomeostasis, oxidative stress…). Importantly, the positive effects of PA might not only arise from the modulation of the amyloid cascade, but the impact on the biology of aging. In fact, PA has been shown to be a core contributor to healthy functional aging, by positively impacting aging-related biology [4,5] (Fig. 1). However, most evidence of the effect of such interventions on AD-related pathophysiology comes from animal models, yielding uncertainty around the extrapolation of findings to humans [6].

**Fig. 1:**
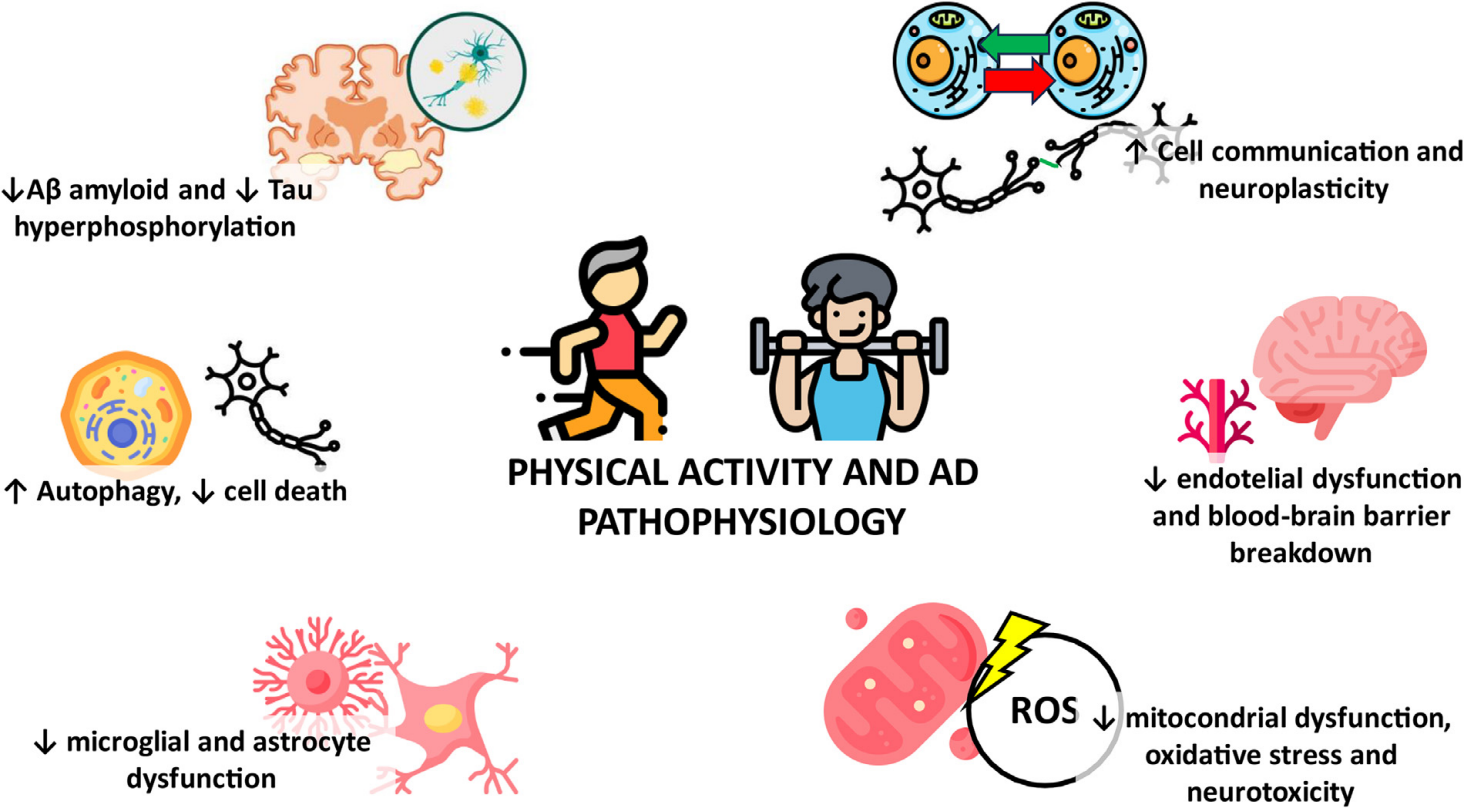
Physical activity and AD Pathophysiology.

Consequently, despite the potential utility of PA/exercise-based interventions in the prevention and management of AD and other dementias, there still exist important gaps in knowledge that might be hampering the incorporation of such interventions to real-world settings. For example, the best type of PA, optimal time to start interventions, or the exact mechanisms by which exercise promotes brain health at advanced ages remains elusive [7]

A recent study by Kim et al. published in this issue of the *Journal of Prevention of Alzheimer’s Disease* investigated the associations between self-reported lifetime walking-based PA and the evolution of markers of AD pathophysiology in the brain in a sample of 151 non-demented older adults (mean age 69.7 ± 7.4, 58.9 % females) over a 4-year follow-up. They assessed cerebral Aβ and tau protein deposition by positron emission tomography, cortical thickness by computerized tomography, and the presence of brain microvascular alterations by means of white matter hyperintensities analysis. Their results pointed that having accumulated higher intensity and greater volumes of walking over time were associated with lower late-life accumulation of Aβ; no benefits for moderate intensity or duration, compared to the lowest PA intensity/duration groups. Furthermore, the age of engagement in walking-related PA determined the associations, with stronger effects among those starting earlier in life. Importantly, no associations were found with other AD-related brain changes, such as tau protein accumulation, cortical thickness or white matter hyperintensities [8].

These results partly reinforce the role of PA as AD-disease protective factor at the pre-clinical disease phase. However, drivers of divergent results with previous studies linking PA AD-related pathophysiologic traits distinct to Aβ need to be highlighted and should be subject of further investigation. Limitations of this study, and the overall bulk of evidence available should be mentioned. Most of previous research exploring links between PA and AD-disease markers relies on self-report of PA, with potential bias due to social desirability and poor recall, especially among older adults. In addition, most studies focused on the role of PA alone, overlooking the role of sedentary behavior, a critical determinant of health that coexists and competes with the latter, but determines differential effects on health. Finally, studies explored the associations/effects of a certain type of PA, either overall, walking or aerobic activities. The exploration of the role of different modes of exercises (aerobic, resistance training) and the incorporation of precision medicine principles, through determination of PA/exercise parameters, such as type, intensity, volume and frequency, that might optimize the effect of the interventions deserves more attention by future research.

In conclusion, the growing number of studies points out that PA/exercise-based intervention might contribute to the prevention/management of AD and other dementias by directly acting on pathophysiological processes of the disease. However, future research focusing on mechanisms underlying such link, as well as the refinement of the interventions at different diseases stages should be incorporated in the future.

## CRediT authorship contribution statement

**Juan Luis Sánchez-Sánchez:** Conceptualization, Supervision, Validation, Writing – original draft, Writing – review & editing. **Philipe de Souto Barreto:** Supervision, Validation, Writing – original draft.

## Declaration of competing interest

The authors declare that they have no known competing financial interests or personal relationships that could have appeared to influence the work reported in this paper.
